# Effect of Metformin on Premature Luteinization and Pregnancy Outcomes in Intracytoplasmic Sperm Injection-Fresh Embryo Transfer Cycles: A Randomized Double-Blind Controlled Trial

**DOI:** 10.22074/IJFS.2020.134643

**Published:** 2021-03-11

**Authors:** Reda S. Hussein, Ihab Elnashar, Ahmed F Amin, Yulian Zhao, Ahmed M. Abdelmagied, Ahmed M. Abbas, Ahmed A. Abdelaleem, Tarek A. Farghaly, Osama S Abdalmageed, Ahmed A. Youssef, Esraa Badran, Hisham A. Abou-Taleb

**Affiliations:** 1 Department of Obstetrics and Gynecology, Faculty of Medicine, Assiut University, Assiut, Egypt; 2 Department of Obstetrics and Gynecology, Mayo Clinic, Rochester, Minnesota, USA; 3 Department of Obstetrics and Gynecology, Taibah University, Medina, KSA

**Keywords:** Infertility, Intracytoplasmic Sperm Injection, Metformin, Pregnancy Outcomes

## Abstract

**Background:**

Premature luteinization (PL) is not unusual in in vitro fertilization (IVF) and could not be wholly avoided by using either gonadotropin-releasing hormone (GnRH) agonists or GnRH antagonist regimens. The study aims to evaluate metformin’s efficacy in preventing PL in fresh GnRH antagonist intracytoplasmic sperm injection (ICSI) cycles with cleavage-stage embryo transfer.

**Materials and Methods:**

This randomized, double-blind, placebo-controlled trial was conducted in a tertiary univer- sity IVF center. We recruited infertile women who were scheduled to perform their first or second ICSI trial. Eligible women were recruited and randomized in a 1:1 ratio into two groups. Metformin was administered in a dose of 1500 mg per day since the start of contraceptive pills in the cycle antecedent to stimulation cycle until the day of ovulation triggering, while women in the placebo group received a placebo for the same regimen and duration. The primary outcome was the incidence of PL, defined as serum progesterone (P) on the triggering day ≥1.5 ng/mL. Secondary outcomes comprised the live birth, ongoing pregnancy, implantation, and good-quality embryos rates.

**Results:**

The trial involved 320 eligible participants (n=160 in each group). Both groups had comparable stimulation days, endometrial thickness, peak estradiol levels, number of oocytes retrieved, and number of mature oocytes. Met- formin group experienced lower level of serum P (P<0.001) and incidence of PL (10 vs. 23.6%, P=0.001). Moreover, lower progesterone/estradiol (P/E) ratio and progesterone to mature oocyte index (PMOI) (P=0.002 and P=0.002, respectively) were demonstrated in women receiving metformin. Metformin group generated a better rate of good- quality embryos (P=0.005) and ongoing pregnancy (43.8 vs. 31.8%, P=0.026). A similar trend, though of borderline significance, was observed in the live birth rate in favor of metformin administration (38.15 vs. 27.5%, P=0.04).

**Conclusion:**

Metformin could be used in patients with potential PL to improve fresh cycle outcomes by preventing PL (Registration number: NCT03088631).

## Introduction

The significance of premature progesterone (P) increase during the late follicular phase, commonly recognized as premature luteinization (PL), as well as its involvement on* in vitro* fertilization (IVF) outcomes has been a subject of intense research (1). So far, it is broadly affirmed that P level ≥1.5 ng/ml on the day of ovulation triggering, reduces the pregnancy rates of fresh embryo transfers (2). PL has been reported to occur in 12.3 to 46.7% of fresh IVF cycles (3). The heterogeneity of methods, cutoffs, or even terminology used to define PL, may explain the wide range reported for PL incidence (4).

The optimal cutoff level of follicular serum P used to define PL is a controversial issue that raises a question which P level has the best prognostic value for IVF success. PL is widely recognized as a rise of serum P≥1.5 ng/ml in the late follicular phase of controlled ovarian stimulation cycles (COS) before ovulation triggering (5). However, several reports have proposed the use of other parameters with higher predictive values for PL impact on IVF cycles such as tailoring P values according to the magnitude of ovarian response (6), progesterone/estradiol ratio (P/E) (5), and recently, progesterone to mature oocyte index (PMOI) (7).

Either gonadotropin-releasing hormone (GnRH) agonists or GnRH antagonist regimens cannot entirely prevent the risk of PL (8). To date, a quality cost-effective approach for the prevention of PL impact on IVF cycles is lacking (1). Embryo freezing with delaying transfer in the subsequent cycles, is commonly practiced to rescue cycles with PL and overcome the PL-induced harm on the endometrium (9). Nevertheless, freezing embryos presents an additional onus on the IVF laboratory in terms of cost and cycle segmentation especially in low resource settings (10). 

Metformin is an insulin-sensitizing agent that improves insulin sensitivity and decreases hyperinsulinemia with a consequent decline of ovarian hyperandrogenism (11). Mansfield et al. (12) elucidated that metformin exhibits an inhibitory effect on the initial step of steroid biosynthesis with a subsequent reduction of P synthesis from the granulosa cells. Metformin hinders steroidogenic acute regulatory protein (STAR) and 3β-hydroxysteroid dehydrogenase (HSD3B) that are crucial in steroidogenesis. Metformin in low doses improved IVF outcomes in nonpolycystic ovarian syndrome (PCOS) patients with previous two or more failed IVF cycles (13).

Our study's hypothesis is that metformin use could improve the ICSI cycle outcomes by reducing the deleterious influence of PL on endometrial receptivity and/or embryo quality. Hence, we performed the first randomized trial to investigate the efficacy of metformin in preventing PL in ICSI cycles.

## Materials and Methods

### Study type, setting, and duration

Study participants were recruited from a single university-affiliated IVF center. This study was a randomized, double-blind, placebo-controlled trial conducted in the period between April, 2017 and March, 2019 (ClinicalTrials. gov: NCT03088631). The study was approved by the Institutional Review Board under number (17200033/2017) and a written informed consent was obtained before the patients’ enrollment. 

### Study participants

Women who pursued their first- or second-ranked ICSI trial at our IVF unit, were counseled for participation. Women aged between 20 and 38 years were included. Patients whose body mass index (BMI) was more than 30 kg/m^2^ , were advised to have 5-10% weight loss through lifestyle modification and exercises for 3 months. We recruited infertile women with anti-Müllerian hormone (AMH) ≥1 ng/ml and day-3 follicle-stimulation hormone (FSH) <10 mIU/ml. All participants had normal levels of prolactin and thyroid-stimulating hormone before starting gonadotropins stimulation. Patients who were known to have diabetes, renal and liver diseases, alcoholism, or drug abuse were excluded. Patients with uterine factor and poor responders, defined according to Bologna criteria (14), were excluded. 

### Sample size calculation

The only study that investigated metformin role in preventing PL, has reported a reduction in PL incidence from 30% in the historic controls to 20% with metformin administration in a cohort of patients irrespective of ovarian reserve markers (10). The sample size was estimated utilizing the OpenEpi program on a statistical significance of 0.05 and power of 85%. The calculated total sample size was 300 women with 150 in each group. After adjusting a dropout rate of 10%, 330 was the total estimated sample.

### Randomization

A statistician performed a computer-generated random table through permuted block randomization method and installed the allocation data in closed opaque envelopes with serial numbers. Each envelope had a card noting the group identifier inside. The statistician kept the key to the allocated group according to the serial numbers until the end of the study. Eligible women, who accepted study’s participation, were assigned randomly in 1:1 ratio to either metformin or placebo groups. The allocation was not changed after it had had been made.

### Study intervention

Women in metformin group received three tablets of metformin 500 mg per day (Cidophage®, Chemical Industries Development Co, Egypt) with the start of contraceptive pills in the preceding cycle until the day of ovulation triggering. The placebo group received three corn flour placebo tablets for the same regimen and period and regimen. Placebo was identical to metformin in size, appearance, and taste. Both study investigator and patients were blinded to the intervention used.

### The* in vitro* fertilization protocol 

When at least three follicles ≥17 mm were encountered, 10,000 IU human chorionic gonadotropin (HCG, Choriomon®, IBSA Pharmaceutical, Egypt) was administered for ovulation triggering. Patients detected to be at risk of moderate to severe ovarian hyperstimulation syndrome (OHSS), were triggered by 0.2 mg sub-cutaneous GnRHa injection (Decapeptyl, Ferring, Germany). Patients serum P and estradiol levels were measured on the day of HCG triggering and analyzed by Mini-Vidas technique with a sensitivity of 0.2 ng/ml (measurement range was 0.2-40 ng/ml)

A transvaginal ultrasound-guided aspiration was done 34 hours after HCG triggering. Matured oocytes were fertilized by ICSI 6 hours after the retrieval with the husband’s sperm. According to the study protocol, 2-3 best cleavage stage embryos were selected for fresh transfer on day 3 after oocyte retrieval. Embryos of good-quality were defined as those achieving eight-cell stage on day 3 with <20 % fragmentation as described by Volpes et al. (15) . Supernumerary embryos of excellent quality were vitrified immediately after the fresh transfer (day 3 after oocyte collection). 

Luteal support with intramuscular P (25 mg twice daily) (Prontogest®, IBSA Pharmaceutical, Egypt) following egg retrieval was standardized for all patients except those triggered with GnRH agonist. Estradiol valerate (2 mg, TID) was added to progesterone for luteal support of cases with agonist trigger. Luteal support was continued till a pregnancy check was performed 14 days after embryo transfer. Quantitative serum beta HCG was repeated after 48 hours in positive pregnancy cases to early predict ectopic pregnancy or pregnancy losses. More than 90 % of the study procedures (ultrasound scanning, oocyte retrieval, and embryo transfer) were carried out by a single researcher.

### Study outcomes

The incidence of PL in both groups was the study's primary outcome. Progesterone ≥1.5 ng/mL was used to diagnose cases of PL. The secondary outcomes included the live birth rate (the number of cases with a living neonate delivered at ≥24 weeks of gestation expressed per 100 initiated cycles), ongoing pregnancy rate (the number of cases with pregnancy ≥12 weeks of gestation expressed per 100 cycles), implantation rate (the percentage of the identified gestational sacs compared to the total number of transferred embryos), and the rate of good-quality embryos formation (defined as the percentage of goodquality day-3 embryos per all two-pronuclear embryos). We assessed the other parameters reported to diagnose the PL, such as the P/E ratio and PMOI. P/E ratio was measured as P (pg/mL) divided by estradiol (pg/mL). PMOI was calculated as the serum P level (ng/ml) divided by the number of mature oocytes.

### Statistical analysis

Collected data were recorded into a Microsoft Access database and analyzed using Statistical Package for Social Science (SPSS Inc., Chicago, Illinois, version 21). We tested the normality of the continuous data utilizing the Shapiro-Wilk test. Normally distributed data are expressed as mean ± standard deviation (SD) and were analyzed by student’s t test. Data that were skewed are presented as median and interquartile range (IQR) and compared by using the MannWhitney test. Chi-square test was used to compare categorical variables. P<0.05 was acknowledged as the level of statistical significance.

## Results

### Baseline demographic and clinical characteristics

**Fig.1:**
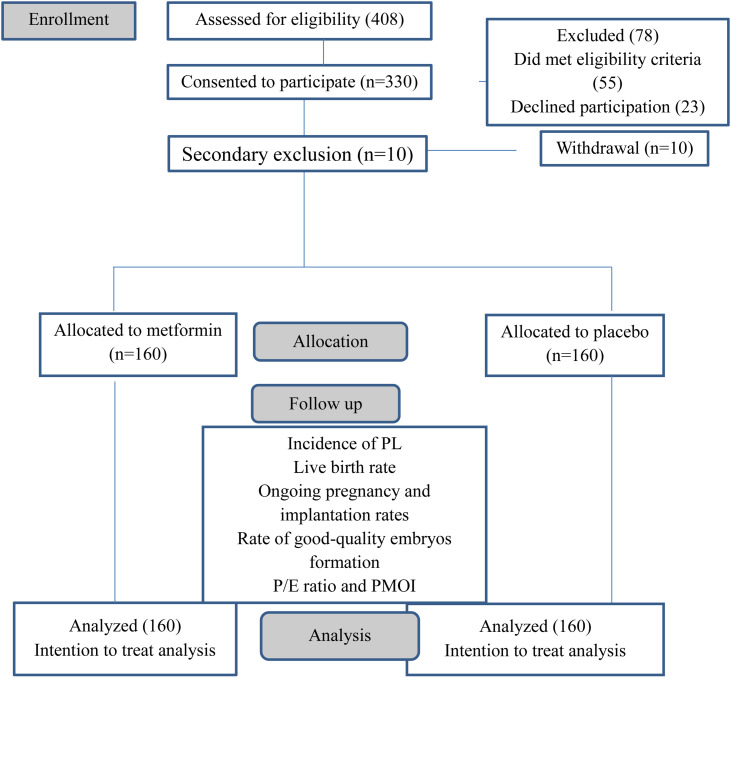
Consort flow diagram of the study. PL; Premature luteinization, P/E; Progesterone/estradiol ratio, and PMOI; Progesterone to mature oocyte index.

Four hundred and eight (408) patients were assessed for eligibility based on the study criteria. Of the total, 55 women did not meet the criteria, and 23 declined participation. After that, we had 10 women who were secondarily excluded due to withdrawal of their consent. The study included 320 patients randomized into two groups of 160 for metformin and 160 for placebo. The CONSORT flow diagram of the study is shown in Figure 1. Three patients did not have fresh embryo transfer in the metformin group [total fertilization failure=2, and severe OHSS=1], whereas the placebo group had 2 cases of total fertilization failure, one case of freeze all due to severe OHSS, and one case of empty follicle syndrome (P=0.446). All patients received HCG trigger except 6 in the metformin group and 8 in the placebo group who received agonist trigger and combined estrogen and progesterone luteal support. Intention-to-treat (ITT) was the method used for data analysis. 

The demographics, including age, BMI, and duration of infertility, were similar (Table 1). The baseline clinical characteristics of both groups are summarized in Table 2. No difference was present regarding etiology and type of infertility (primary or secondary), the rank of ICSI cycle, AMH, FSH, and luteinizing hormone (LH) levels. The metformin group had a higher antral follicle count (AFC) [median (IQR): 13(8) vs. 11(5), P<0.005]. Nevertheless, this difference was not clinically significant as both groups were within the range of anticipated normal ovarian responders. Table S1 (See Supplementary Online Information at www.ijfs. ir) shows patients with BMI ≥30 Kg/m^2^ who underwent a trial of weight reduction before study participation in both study groups.

**Table 1:** The demographic characteristics of the study participants


Variables	Metformin groupn=160	Placebo groupn=160	P value

Age (Y)	29.9 ± 3.5	30.8 ± 4.5	0.054^a^
BMI (Kg/m^2^)	28.6 (6)	28 (5)	0.166^b^
Duration of infertility (Y)	6.2 ± 2.6	6.7 ± 3.5	0.115^a^


Data are presented as mean ± SD or median (IQR).^ a^ ; Student's t test, ^b^; Mann-Whitney test, and BMI; Body mass index.

**Table 2:** The baseline clinical characteristics of the study participants


Variables	Metformin group n=160	Placebo group n=160	P value

Etiology of infertility			
Male factor	59 (36.8)	54 (33.7)	0.215^a^
Unexplained	31 (19.4)	48 (30.0)	
Ovulatory disorders	28 (17.5)	27 (16.9)	
Tuboperitoneal factors	23 (14.4)	20 (12.5)	
Combined factors	19 (11.9)	11 (6.9)	
Rank of ICSI cycle			
First ICSI cycle	137 (85.6)	132 (82.5)	0.376^a^
Second ICSI cycle	23 (14.4)	28 (17.5)	
Type of infertility			
Primary infertility	109 (68.1)	115 (71.9)	0.443^a^
Secondary infertility	51 (31.9)	45 (28.1)	
AFC	13 (8)	11 (5)	0.005^b^
AMH level (ng/ml)	3.6 ± 2.2	3.1 ± 3.4	0.10^c^
Basal FSH (mIU/ml)	5.5 ± 2.1	6.0 ± 2.4	0.09^c^
Basal LH (mIU/ml)	4.9 ± 2.8	4.3 ± 2.7	0.083^c^
Basal estradiol	25.7 ± 14.7	30.2 ± 10.3	0.294^c^


Data are presented as number and %, median (IQR) or mean ± SD. Significant P value is presented in bold (P<0.05). ^a^; Chi-square test, ^b^; Mann-Whitney test, ^c^; Student's t test, ICSI; Intracytoplasmic sperm injection, AFC; Antral follicle count, AMH; Anti-Müllerian hormone, FSH; Follicle-stimulating hormone, and LH; Luteinizing hormone.

#### Cycle stimulation parameters

The metformin group received a lower total dose of the gonadotropins used [2700 (1275) vs. 3300 (1425) IU, P<0.001] but with similar stimulation days in comparison to the placebo group. After adjustment of AFC, the metformin group still has a lower total gonadotropins dose (P<0.001). Both groups were comparable in endometrial thickness, endometrial pattern, and number of follicles ≥15 mm on day of HCG triggering. The triggering day P level was significantly reduced in the metformin group than that of the placebo group [0.9 (0.5) vs. 1.1 (0.7), P<0.001, Table 3].

#### Treatment outcomes

Table 4 compares the reproductive outcomes between the study groups. The number of mature oocytes and oocyte maturation index were homogenous in both groups. Although the number of fertilized oocytes was higher in the metformin group (P=0.02), the oocyte fertilization rate did not have a significant difference (P=0.215). Women treated with metformin showed a higher number (P<0.001) and rate (P=0.005) of good-quality embryos. The metformin group had lower level of serum P (P<0.001), P/E ratio (P=0.002) and PMOI (P=0.002). The metformin group also experienced a lower incidence of PL, defined as serum P ≥1.5 ng/ml (10.0 vs. 23.6%, P=0.001). Metformin administration resulted in higher implantation and ongoing pregnancy rates (22.3 vs. 15.8%, P=0.026; 43.8 vs. 31.8%, P=0.02, respectively). Metformin administration achieved a significant rise, though of borderline significance, in live birth rate [61/160 (38.1%) vs. 44/160 (27.5%), P=0.04].

**Table 3:** The cycle clinical data before oocyte retrieval


Variables	Metformin group n=160	Placebo group n=160	P value

Stimulation days	11.7 ± 1.4	11.7 ± 1.5	0.860^a^
Total gonadotropins dose	2700 (1275)	3300 (1425)	<0.001^b^
Peak estradiol (pg/ml)	3330.8 ± 2020.8	3106.7 ± 1988.5	0.317^a^
Progesterone on triggering day (ng/ml)	0.9 (0.5)	1.1 (0.7)	<0.001^b^
Endometrial thickness (mm)	10.7 ± 1.5	10.5 ± 1.8	0.277^a^
Number of follicles ≥15 mm	17 (11)	16 (10)	0.096^b^
Endometrial pattern			
Pattern A	134 (83.8)	135 (84.4)	0.420^c^
Pattern B	22 (13.7)	18 (11.2)	
Pattern C	4 (2.5)	7 (4.4)	
Trigger type and luteal support			
HCG+P	154 (96.3)	152 (95)	0.523^c^
GnRH agonist+P and E	6 (3.7)	8 (3)	
Fresh transfer			
Yes	157 (98.1)	158 (97.5)	0.446^c^
No	3 (1.9)	2 (2.5)	
Severe OHSS	1 (0.63)	1 (0.63)	
Empty follicles	0 (0)	1 (0.63)	
Fertilization failure	2 (1.25%)	2 (1.25%)	


Data are presented as mean ± SD, median (IQR) or number and %. Significant P value is presented in bold (P<0.05). ^a^; Student's t test, ^b^; Mann-Whitney test, ^c^; Chi-square test. Endometrial pattern: Pattern A; A triple-line pattern, Pattern B; An intermediate isoechogenic, Pattern C; Homogenous hyper-echogenic endometrium, HCG; Human chorionic gonadotropin, GnRH; Gonadotropinreleasing hormone, P; Progesterone, E; Estrogen, and OHSS; Ovarian hyperstimulation syndrome.

**Table 4:** Treatment outcomes


Variables	Metformin group n=160	Placebo group n=160	P value

Retrieved oocytes	15 (10)	14 (11)	0.084^a^
Mature oocytes	12 (8)	10 (9)	0.103^a^
Oocyte maturation index	75.7 ± 15.7	73.7 ± 17.9	0.288^b^
Fertilized oocytes	9 (6)	7 (8)	0.021^a^
Fertilization rate	75.9 ± 19.1	73.1 ± 21.8	0.215^b^
Number of good quality embryos	5.2 ± 2.7	4.1 ± 2.6	<0.001^b^
Rate of good-quality embryos formation	60.0 (16.7)	50.0 (26.7)	0.005^a^
Embryo cryopreservation			
Yes	122 (76.3)	83 (51.9)	<0.001^c^
No	38 (23.7)	77 (48.1)	
Transferred embryos	2.5 ± 0.7	2.4 ± 0.8	0.434^b^
Incidence of PL (P≥1.5 ng/ml)	16/160 (10.0)	38/160 (23.8)	0.001^c^
P/E ratio	0.31 (0.2)	0.41 (0.3)	0.002^a^
PMOI	0.08 (0.07)	0.11 (0.1)	0.002^a^
Implantation rate (%)	22.3	15.8	0.026^c^
Ongoing pregnancy rate	70/160 (43.8)	51/160 (31.8)	0.026^c^
Live birth rate	61/160 (38.1)	44/160 (27.5)	0.04^c^


Data are presented as median (IQR), mean ± SD or number and %. Significant P value is presented in bold (P<0.05). ^a^ ; Mann-Whitney test, ^b^; Student's t test, ^c^ ; Chi-square test, PL; Premature luteinization, P; Progesterone, P/E; Progesterone/estradiol, and PMOI; Progesterone to mature oocyte index.

## Discussion

The current study demonstrated that metformin administration before and during IVF stimulation reduces the preovulatory serum P levels and improves implantation and ongoing pregnancy rates, possibly by preventing the putative deleterious effect of PL on endometrium receptivity and embryo quality. Metformin reduced the incidence of PL based on the absolute level of serum P, P/E ratio, and PMOI. These effects were reflected by a significant, though borderline, rise of live birth rate with metformin administration.

PL is not an uncommon IVF problem that was reported in all profiles of patients undergoing COS, and no IVF cycle was found to be immune from it (1, 11). Various measures were introduced to lessen the risk of PL in assisted reproductive technology (ART): i. Supplementing corticosteroids to the conventional stimulation protocol in cases with higher basal P (16), ii. Proper timing of ovulation triggering (17), iii. Step-down dose regimen to avoid the intense ovarian stimulation toward the final days of oocyte maturation (18), iv. Use of aromatase inhibitors (19), and v. Metformin (10). Nevertheless, all the aforementioned strategies are in need of further welldesigned trials to establish their efficacy in preventing PL in COS.

It was proposed that PL negatively influences IVF outcomes by generating endometrial advancement that hinders the necessary synchrony for embryo development on the endometrium (20). This analysis was driven by the variation observed in endometrial gene expression profiles between patients with and without P elevation (21). 

Our data revealed that metformin improved the rate of good-quality embryos formation, probably through decreasing PL. However, the influence of PL on embryo and oocyte quality is still disputable in contrary to its unquestioned hostile effect on endometrial receptivity (1). Several authors proposed that PL is related neither to embryo nor to oocyte quality, based on data from egg donation cycles and favorable outcomes after the transfer of frozen-thawed embryos arising from cycles with high P (22). Nonetheless, there is expanding evidence regarding the potentially deleterious impact of PL on the number of good-quality embryos in the different ovarian response categories (23).

In a large retrospective study of 4,651 patients who pursued their first IVF trial, PL was correlated with reduced top-quality embryo rate and cumulative live births, despite higher retrieved oocytes, regardless of the magnitude of ovarian response (24). Likewise, the topquality blastocyst formation rate was inversely associated with P levels on the HCG triggering day in 4,236 fresh GnRH antagonist cycles (25). These results concur with the data of Vanni et al. (26) that showed disturbed embryo quality with increasing P levels. A recent study reviewed the impact of PL on the cumulative live birth and rate of embryo utilization. Patients complicated with PL experienced a significantly lower embryo utilization rate for both blastocyst and cleavage stages (23).

Manno and Tomi (10) were the first to propose the effect of metformin on PL in ICSI cycles. Metformin was administered in a dose of 1000-1500 mg/day from the day of the first ultrasound cycle monitoring until ovulation triggering and compared the cohort of patients received metformin to a group of historic controls. Metformin administration resulted in a significant decrease of PL and increase of pregnancy rate irrespective of the patients’ ovarian reserve.

Jinno et al. (27) reported that low-dose metformin improves implantation and pregnancy rates in non-PCOS repeaters compared with previous IVF trials without metformin. A worldwide web-based survey found that 70% of IVF cycles proposed enhanced pregnancy rate and diminished miscarriage rate with metformin (28). A retrospective study elucidated that metformin might increase pregnancy in non-PCOS patients by improving oocyte and embryo quality in IVF cycles and increasing the number of retrieved oocytes and embryos available for transfer and cryopreservation (29). 

The effect of metformin in IVF cycles was extensively studied in PCOS patients and showed conflicting data. Previous studies indicated that metformin has an improving effect on pregnancy and live birth rates in PCOS (30). In contrary, a recent cochrane review showed a non-significant effect for metformin on the live birth rate despite increasing clinical pregnancy rate (31). 

Clinicians should vigilantly anticipate stimulation cycles at a higher risk of PL. Various factors were reported to contribute to a raised risk of PL, such as a history of recurrent implantation failure (32), higher daily FSH dose (33), higher dose of gonadotropins used (34), and more stimulation days (35). Higher follicular P levels were associated with an increase in oocytes retrieved or elevated estradiol levels (33). The exclusive use of recombinant FSH without LH is related to an increased incidence of PL (36).

The principal strength of the current study is its design, in addition to the novel rationale. This is the first prospective randomized trial performed to investigate the metformin’s role in preventing PL in ART. The incidence of PL was assessed according to not only the absolute P cut-off level (primary outcome) but also the P/E ratio and PMOI. The study outcomes extended to have live birth rate in addition to other pregnancy and embryological outcomes.

The study was not free of limitations. This work lacks investigating a marker for endometrial receptivity to reflect the impact of PL in the studied women. Our study standardized the fresh transfer of cleavage stage embryos. Although the morphological quality criteria of blastocysts have a better predictive value (37), the number of good-quality cleavage stage embryos was reported as an appropriate prognostic tool for live birth, pregnancy and implantation rates in IVF/ICSI cycles (38). The relatively high BMI of the included participants may represent a limitation for generalizability of data and underrepresentation of under- or average- weight profile. Yet, this is the average representative BMI of infertility cohort in the study settings. Further studies areneeded to test the efficacy of metformin use in each ovarian response category.

## Conclusion

Our current trial suggests the use of metformin in cases with potential PL in order to improve pregnancy outcomes in fresh ICSI cycles by ameliorating the impact of PL on either endometrial receptivity and/or embryo quality. This study should be endorsed by a larger randomized trial with more accurate assessment of endometrial receptivity and oocyte/embryo quality

## Supplementary PDF


