# Supplementary material for: Effect of Metformin on Premature Luteinization and Pregnancy Outcomes in Intracytoplasmic Sperm Injection-Fresh Embryo Transfer Cycles: A Randomized Double-Blind Controlled Trial
Source: Int J Fertil Steril. 2021 Mar 11;15(2):108–14. doi: 10.22074/IJFS.2020.134643 (PMC8052800; doi:10.22074/IJFS.2020.134643)
Supplement: Supplementary file 1 [file Int-J-Fertil-Steril-15-108-s01.pdf]

Int J Fertil Steril
International Journal of Fertility & Sterility
ISSN:2008-076X eISSN:2008-0778

Supplementary Information for

Effect of Metformin on Premature Luteinization and Pregnancy Outcomes in Intracytoplasmic Sperm Injection-Fresh Embryo Transfer Cycles: A Randomized Double-Blind Controlled Trial

Reda S. Hussein, M.D.[1, 2*], Ihab Elnashar, M.D.[1], Ahmed F Amin, M.D.[1], Yulian Zhao, M.D., Ph.D.[2], Ahmed M. Abdelmagied, M.D.[1, 3], Ahmed M. Abbas, M.D.[1], Ahmed A. Abdelaleem, M.D.[1], Tarek A. Farghaly, M.D.[1], Osama S Abdalmageed, M.D.[1], Ahmed A. Youssef, M.D.[1], Esraa Badran, M.D.[1], Hisham A. Abou-Taleb, M.D., Ph.D.[1]

1. Department of Obstetrics and Gynecology, Faculty of Medicine, Assiut University, Assiut, Egypt
2. Department of Obstetrics and Gynecology, Mayo Clinic, Rochester, Minnesota, USA
3. Department of Obstetrics and Gynecology, Taibah University, Medina, KSA

Tables 1: Initial and post-weight reduction BMI of participants with BMI ≥30 Kg/m^2

Weight reduction group	Metformin n=61	Placebo n=54
BMI before weight reduction (Kg/m^2)	34.0 (1.18)	33.7 (2.1)
BMI after weight reduction (Kg/m^2)	32.1 (0.75)	31.67 (2.1)

Data are presented as median (IQR). BMI; Body mass index.

Received: 3 May 2020, Accepted: 14 September 2020
*Corresponding Address: Department of Obstetrics and Gynecology, Faculty of Medicine, Assiut University, Assiut, Egypt

Email: rsalah313@yahoo.com
